# A Fusion-Assisted Multi-Stream Deep Learning and ESO-Controlled Newton–Raphson-Based Feature Selection Approach for Human Gait Recognition

**DOI:** 10.3390/s23052754

**Published:** 2023-03-02

**Authors:** Faiza Jahangir, Muhammad Attique Khan, Majed Alhaisoni, Abdullah Alqahtani, Shtwai Alsubai, Mohemmed Sha, Abdullah Al Hejaili, Jae-hyuk Cha

**Affiliations:** 1 Department of Computer Science, HITEC University, Taxila 47080, Pakistan; 2 Department of Informatics, University of Leicester, Leicester LE1 7RH, UK; 3 College of Computer Science and Engineering, University of Ha’il, Ha’il 81451, Saudi Arabia; 4 Department of Software Engineering, College of Computer Engineering and Sciences, Prince Sattam bin Abdulaziz University, Al-kharj 16242, Saudi Arabia; 5 Department of Computer Science, College of Computer Engineering and Sciences, Prince Sattam bin Abdulaziz University, Al-kharj 16242, Saudi Arabia; 6 Faculty of Computers & Information Technology, Computer Science Department, University of Tabuk, Tabuk 71491, Saudi Arabia; 7 Department of computer Science, Hanyang University, Seoul 04763, Republic of Korea

**Keywords:** gait recognition, contrast enhancement, deep learning, feature selection, fusion, machine learning

## Abstract

The performance of human gait recognition (HGR) is affected by the partial obstruction of the human body caused by the limited field of view in video surveillance. The traditional method required the bounding box to recognize human gait in the video sequences accurately; however, it is a challenging and time-consuming approach. Due to important applications, such as biometrics and video surveillance, HGR has improved performance over the last half-decade. Based on the literature, the challenging covariant factors that degrade gait recognition performance include walking while wearing a coat or carrying a bag. This paper proposed a new two-stream deep learning framework for human gait recognition. The first step proposed a contrast enhancement technique based on the local and global filters information fusion. The high-boost operation is finally applied to highlight the human region in a video frame. Data augmentation is performed in the second step to increase the dimension of the preprocessed dataset (CASIA-B). In the third step, two pre-trained deep learning models—MobilenetV2 and ShuffleNet—are fine-tuned and trained on the augmented dataset using deep transfer learning. Features are extracted from the global average pooling layer instead of the fully connected layer. In the fourth step, extracted features of both streams are fused using a serial-based approach and further refined in the fifth step by using an improved equilibrium state optimization-controlled Newton–Raphson (ESOcNR) selection method. The selected features are finally classified using machine learning algorithms for the final classification accuracy. The experimental process was conducted on 8 angles of the CASIA-B dataset and obtained an accuracy of 97.3, 98.6, 97.7, 96.5, 92.9, 93.7, 94.7, and 91.2%, respectively. Comparisons were conducted with state-of-the-art (SOTA) techniques, and showed improved accuracy and reduced computational time.

## 1. Introduction

Human verification or identification plays a significant role in information security, public security systems, point-of-sales machines, automatic teller machines, etc. [1]. Human beings can be identified by examining their different external and internal body parts, such as blood samples, skin, hair, ear shape, bite by forensic odontology, face recognition, and walking style by gait analysis [2]. Fingerprints and face recognition are well-known biometric systems, but both have some limitations; fingerprint verifications need contact with fingers, and face recognition needs a controlled environment and proper distance.

On the other hand, human gait analysis [3] is an application for recognizing people at a certain distance; basically, gait refers to the individual walking style of human beings [4]. Mainly, there are two approaches to gait recognition: model-based and model-free. The model-based approach tracks parts of the human body, such as arms, legs, hands, feet, and neck, and this approach obtains a set of static and dynamic parameters [5]. The basic idea behind this approach is to model the human skeleton’s bones and joints. Furthermore, the model-free process tracks an object’s geometry and shapes, which is helpful in object recognition systems [6].

Gait recognition is widely used in many applications, such as medical sciences [7], personal recognition, sports sciences, and cyber security. The human gait has 24 elements that can be used to identify a person. It is proven in different studies and experiments that every person has a unique muscular-skeletal structure that shows that it is possible to recognize a person with the help of gait information [8]. Gait recognition has more magnificent characteristics than other biometrics identifiers [9]. Firstly, the human gait can be captured far away without the subject’s cooperation [10], while all other biometrics cannot be obtained without the person connecting physical and mental involvement with data-acquiring sensors [11,12]. Secondly, gait recognition can be performed on low-resolution images or videos, while other biometrics, such as facial recognition, cannot be performed well on low-resolution images or videos [13]. Thirdly, HGR can be performed with minimal equipment, such as a camera, accelerometer, floor sensor, and radar [14].

Most gait recognition systems are based on four main components [15]. The first is capturing gait data from video sequences and real-time scenarios using different tools and techniques [16]. The second one is to apply segmentation to identify the human body shape and remove noise and blueness from the background by using different segmentation techniques based on the different characteristics of an image and region of interest. The third one is contour detection, which will be needed when a gap occurs between joins or some missing part of a human body after the segmentation. Contour detection is useful for the analysis of shape and object detection. The last one is feature extraction based on human properties, such as shape, geometry, etc. These features are finally classified using machine learning classifiers [17].

Applications in image processing require improving each image or frame before proceeding to the next level, such as feature extraction. The enhancement methodology relies on high-frequency pixels and low-frequency components [18]. High-frequency pixel components depict the scene and objects in the image, whereas low-frequency pixel components indicate minute features, including certain lines and minuscule points. To enhance the image, great attention is always required to increase the high-frequency components while retaining the low-frequency components [19]. Recently, deep learning has shown significant success in object classification, gait recognition, action recognition, amongst others. In deep learning, features are extracted from the raw images. A deep learning architecture consists of several hidden layers: convolutional, pooling, and fully connected. Training the deep learning model on the enhanced dataset may yield better features that later improve accuracy [20].

### 1.1. Existing Techniques

Several deep learning-based techniques for human gait classification have been introduced in the literature. Muhammad et al. [21] implemented an improved ant colony optimization (IACO) and deep learning framework for human gait recognition. The IACO algorithm has been used to select the best-classified features using machine learning classifiers. After several experimentations, the IACO method is more accurate while diminishing the computational cost compared to the other state-of-the-art techniques. Imran et al. [22] presented a deep learning (DL) and kurtosis-controlled entropy (KcE) based framework for HGR using video sequences. They tried to resolve challenges, such as human angle shift, clothing, and walking style. The authors extracted features using ResNet101 deep model and then selected the best features using the KcE approach. The experimental process was conducted on the CASIA-B dataset and obtained an accuracy of 95.26% and 96.60%, respectively. Khan et al. [23] introduced a single-stream HGR framework based on optimal deep learning fused features. In their work, the authors performed data augmentation at the first step and then used two pre-trained models, such as Inseption-ResNet-V2 and NASNet mobile. Features of both deep learnings were fused and further optimized using the whale optimization algorithm. Machine learning classifiers were applied and obtained the best accuracy of 89%.

Huang et al. [24] demonstrated a gait recognition method based on multisource sensing information. The 3D human features data was extracted using the human body’s structure and multisource stream information during a human walk. Athlete walk includes different characteristics, and based on these characteristics, a person is identified. The CASIA A dataset was used for the experimental process and obtained an accuracy of 88.33%. Hasan et al. [25] presented a modified residual block and a novel shallow convolutional layer for HGR. Wearable sensors were embedded in objects that can be worn on the subject body, such as wristwatches, necklaces, and smartphones, and were used for gait analysis. Template matching and conventional matching were not appropriate and did not provide improved performance for low-device wearable devices. They also introduced a modified residual block and shallow convolutional neural network that obtained an accuracy of 85% on the IMU-based dataset. Junaid et al. [26] presented a human gait analysis approach for osteoarthritis using DL and kernel extreme learning machine. The authors faced numerous difficulties in this approach, such as abnormal walking, patients’ clothes, and angle changes. Conventional techniques are only concerned with feature selection and do not address such issues; therefore, the authors employed a novel robust method to address that disparity. For experimental purposes, two pre-trained models (VGG16-Net and AlexNet) were used and obtained improved accuracy. Yonghong et al. [27] addressed the free-view gait recognition problem. They faced the problems of traditional methods that capture gait sequences under uncontrolled scenes, unknown view angles, and dynamically changing viewing angles during the walk. They presented a unique walking trajectory fitting (WTF) approach for these challenges. Also, they introduced a joint gait manifold (JGM) technique for gait similarity evaluation.

### 1.2. Major Challenges

In summary, all the above methods still faced several issues, such as selecting important features and extracting irrelevant features. Moreover, the above methods did not select the entire CASIA-B dataset for the experimental process. Features extraction from the original video frames may extract some redundant and irrelevant features due to complicating factors, such as outdoor environment, lighting conditions, complex background, noise, and low-resolution frames. These factors impact recognition accuracy. In addition, several studies extracted features from a region of interest (ROI), which is a time-consuming step that, sometimes, leads to a chance of incorrect ROI detection. The incorrect ROI detection consumes the developed system’s overall time and extracts irrelevant features that later reduce the classification accuracy. Therefore, this work presents a new framework using the best fusion-assisted deep learning features.

### 1.3. Major Contributions

The major contributions of this work are as follows:

- A contrast enhancement technique based on local and global filter information fusion is proposed. The high-boost operation is finally applied to highlight the human region in a video frame.
- The data augmentation was performed, and two fine-tuned deep learning models (MobilenetV2 and ShuffleNet) were trained using deep transfer learning. Features were extracted from the flattened global average pooling layers instead of the fully connected layer.
- Features of both streams were fused in a serial-based fashion that can minimize the loss of information and then select the best features using a new approach called ESO-controlled Newton–Raphson.
- The detailed ablation study-based results have been computed and discussed, showing the improvement in the accuracy of this work.

### 1.4. Manuscript Organization

The rest of the manuscript is organized in the following order. The proposed methodology is presented in Section 2, which includes the contrast enhancement technique, deep learning features, proposed feature fusion, and best feature selection. Section 3 presents the experimental results of the proposed methodology. Section 4 concludes the manuscript.

## 2. Proposed Methodology

**Figure 1:**
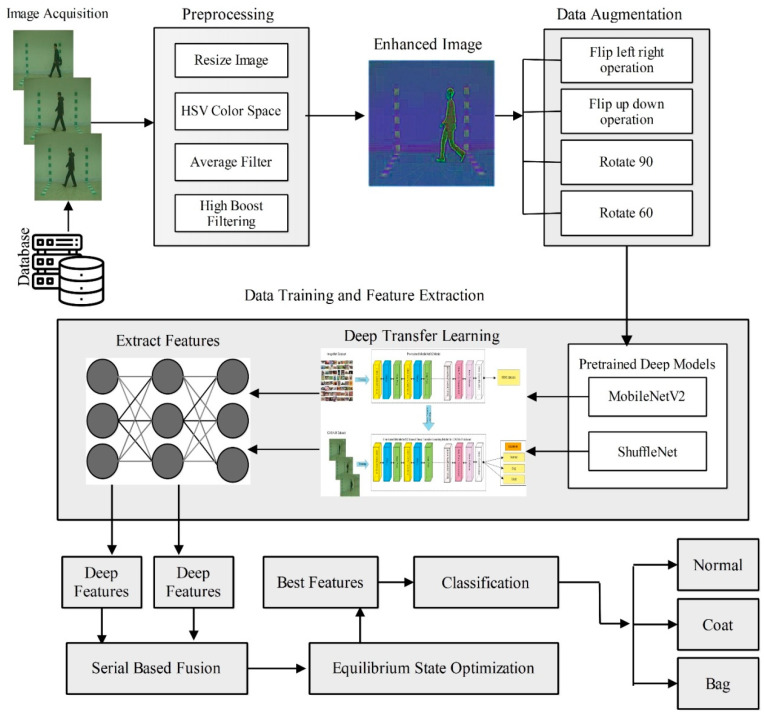
Proposed framework of HGR using two-stream fusion-assisted deep learning and optimal features selection.

The proposed human gait recognition framework is presented in Figure 1. This figure illustrates that the proposed HGR framework consists of several phases: contrast enhancement of original frames, data augmentation, training of the deep learning models, extraction of features, a fusion of both stream features, selection of the most optimal features, and finally, classification. A brief description of each step in the form of mathematics and numerical values is discussed below.

### 2.1. Novelty 1: Hybrid Fusion Enhancement Technique

Preprocessing is an important step in computer vision that can be used to refine the original images into better information. In this work, a new fusion-based technique is proposed for contrast enhancement and improvement of frame quality. For this purpose, HSV color transformation is initially applied, which encodes 24-bit colors by hue, saturation, and value. This selection aims to organize the colors in a more practically applicable manner. Consider we have a CASIA-B dataset denoted as ẞ and ẞ ℇ ℛ. ẞ (i, j) denotes the HSV color-transformed image, and R, G, and B denote the red, green, and blue channels of values between 0–255. The mathematical formulation is defined as follows:

(1) ℟=R/255

(2) Ꞡ=G/255

(3) ₿=B/255

(4) RangMax=max (℟,Ꞡ,₿)₿=B/255

(5) ℟RangMin=min (℟,Ꞡ,₿)

(6) Ꝿ=RangMAX−RangMin

Here Ꝿ denotes the change in maximum and minimum range, ℟ denotes the extracted red channel, Ꞡ denotes the extracted green channel, and ₿ denotes the extracted blue channel, respectively. Based on the above information, the hue, saturation, and value channels are computed as follows:

(7) $$H=\left\{\begin{array}{c} 60{}^{\circ}\times \frac{\left(Ꞡ-₿\right)}{Ꝿ}\;mod\;6,\;RangMax=℟\\ 60{}^{\circ}\times \frac{\left(₿-℟\right)}{Ꝿ}+2,\;RangMax=Ꞡ\\ 60{}^{\circ}\times \frac{\left(℟-Ꞡ\right)}{Ꝿ}+4,\;RangMax=₿ \end{array}\right\}$$

(8) $$S=\left\{\begin{array}{c} 0,\;RangMax=0\\ \frac{Ꝿ}{RangMax},\;RangMax\;\neq 0\; \end{array}\right\}$$

(9) V=RangMax

After that, the averaging filter is applied to the transformed image due to its linear filter type. This filter minimized the ambient noise, refined edges, and rectified uneven lighting. This approach involves filtering the frame by correlation with a suitable filter kernel. The value of the resultant pixel is determined as the weighted combination of its neighbor pixels. On the input signal, it functions as an averaging filter; it processes an input vector of values and determines an average for every value inside the vector. Consider we have an input image Ɪ (u, v) filtered image Ꞇ (u, v), and ґ (a, b) is the weight of the average filter that convolves the input image and produced the smooth filtered image: the mathematically averaging filter can be illustrated as:

(10) $$Ꞇ\;\left(u,\;v\right)=\frac{\left(\sum_{a=-u}^{x}\sum_{b=-v}^{y}Ύ\;\left(\;a,\;b\right).Ɪ\left(u,\;v\right)\right)}{\sum_{a=-u}^{x}\sum_{b=-v}^{y}Ύ\;\left(a,\;b\right).\;}$$

**Figure 2:**
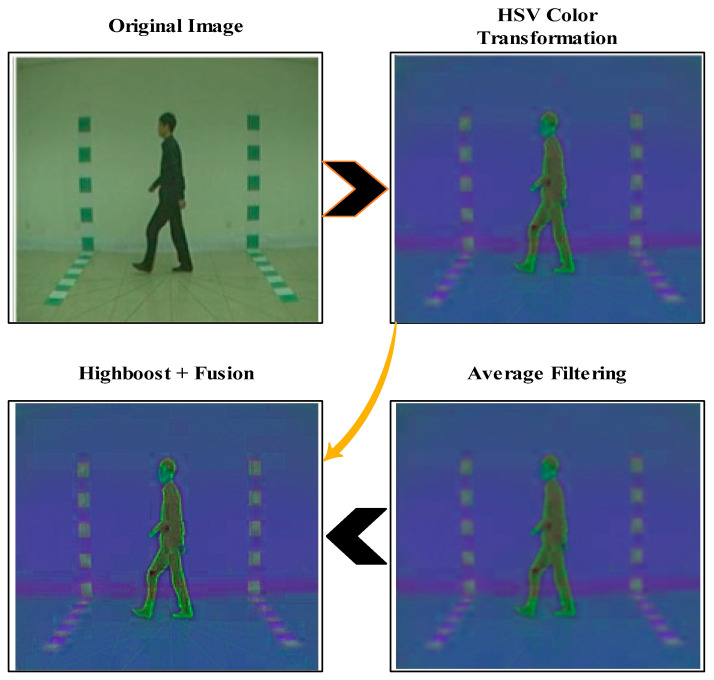
Proposed fusion of filters for contrast enhancement of moving subject.

The high-boost filter is later applied on the resultant image Ꞇ (u, v) for the sharpening of edges in a video frame. This operation is employed to strengthen the image of high-frequency components, further improving the relative relevance of features conveyed by high-frequency components. The high-boost filtering image is computed as follows:

(11) $$P_{hb}\left(u,v\right)=\left(W-1\right)*Ꞇ\;\left(u,\;v\right)+Ꞇ\;\left(u,\;v\right)*h_{hpf}\left(a,b\right)$$

(12) $$\tilde{P_{hb}}\left(u,v\right)=[P_{hb}\left(u,v\right)+ẞ\;\left(i,\;j\right)]-I\left(u,v\right)]$$

where W is an increasing factor for adjusting the weights, $P_{hpf}\left(u,v\right)$ denotes the high-pass filtered image, and $\tilde{P_{hb}}\left(u,v\right)$ is the final fused enhanced image. The visual illustration is shown in Figure 2. Based on these contrast enhancement outputs, we augmented the entire dataset and results outputs are described in Table 1.

### 2.2. Pre-Trained Deep Models

**Figure 3:**
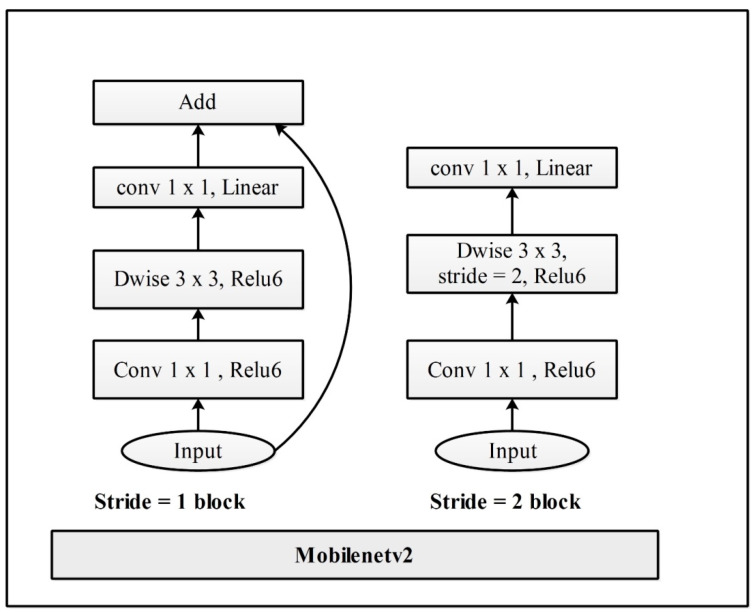
Original bottleneck architecture of MobilenetV2.

Mobilenetv2: Mobilenetv2 was introduced by Google in 2018, and is the variation of the Mobilenet model. It is a convolutional neural network that contains 53 deep layers. This model is based on the inverted residual structure with connections between the bottleneck levels. Therefore, we used this model as a backbone network. A visual illustration of this network is shown in Figure 3 In this figure, it is noted that there are two blocks. One is a residual block with a stride of 1. Another one is a block with a stride of 2 for downsizing. There are three layers for both types of blocks. This time, the first layer is 1 × 1 convolution with ReLU6. The second layer is the depth-wise convolution. The third layer is another 1 × 1 convolution but without any non-linearity. It is claimed that if Relu is used again, the deep networks only have the power of a linear classifier on the non-zero volume part of the output domain. It is a very effective feature extractor mostly used for object detection and segmentation. Mobilenetv2 was pre-trained using the ImageNet dataset of about 1000 object classes.

**ShuffleNet:** The ShuffleNet is a lightweight convolutional neural network (CNN) model introduced by Magvii Inc in 2017. It is a 50-layer deep lightweight CNN model with 1.4 million parameters and accepts an RGB input image of a size of 224 × 224. They proposed a CNN model suitable and specially designed for mobile devices, which is highly efficient in computation and power consumption. This model is evaluated in ImageNet 2016 classification dataset. It introduces the three variants of the shuffle unit, composed of group convolution and channel shuffles. Group convolution uses multiple kernels per layer and generates multiple channel outputs per layer. It can learn more intermediate features and increase the channels for the next layer.

**Figure 4:**
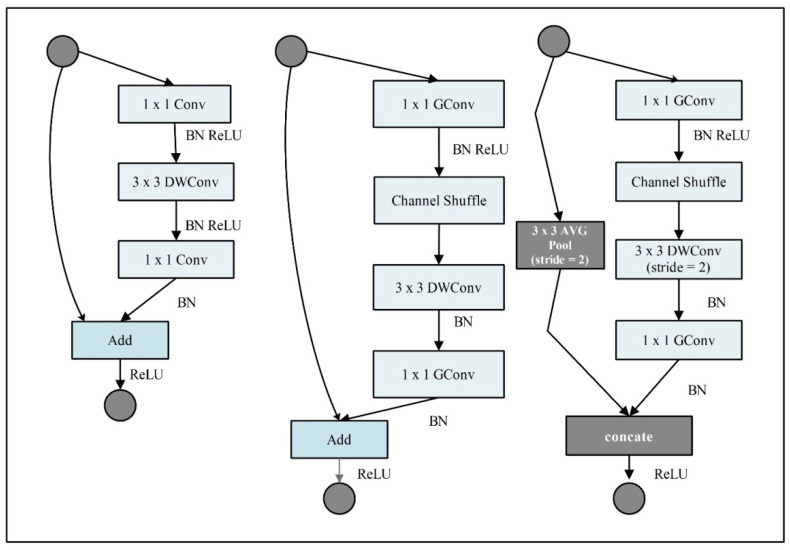
Original architecture of ShuffleNet deep model.

Moreover, channel shuffle is an operation to help information flow across feature channels in a CNN. It was used as a part of the ShuffleNet architecture. The input and output channels can be fully related if a group convolution is allowed to obtain input data from different groups. It can be designed with very limited computing power. Two new operations are used in this architecture (pointwise group convolution and channel shuffle) that reduce the computation cost while maintaining accuracy. The visual description of this architecture is shown in Figure 4.

### 2.3. Deep Transfer Learning based Features Extraction

The deep transfer learning process is employed in this work for the training of both pre-trained models on the enhanced CASIA-B dataset. For the deep transfer learning process, we firstly fine-tuned both models so that the last three consecutive layers were removed, and new layers were added and connected to the previous global average pooling layer. Next, the models were trained through deep transfer learning.

**Domain.** A domain can be represented as:

(13) D={V, ρ (V)} 

which contains two parts: the feature space, V, and the probability distribution, ρ(V). V={v | vi ℇ V, i=1,………,N } and N is a dataset with N instances. However, source and target domains are the subcategory of transfer learning with the same feature vector but different probability distributions.

**Figure 5:**
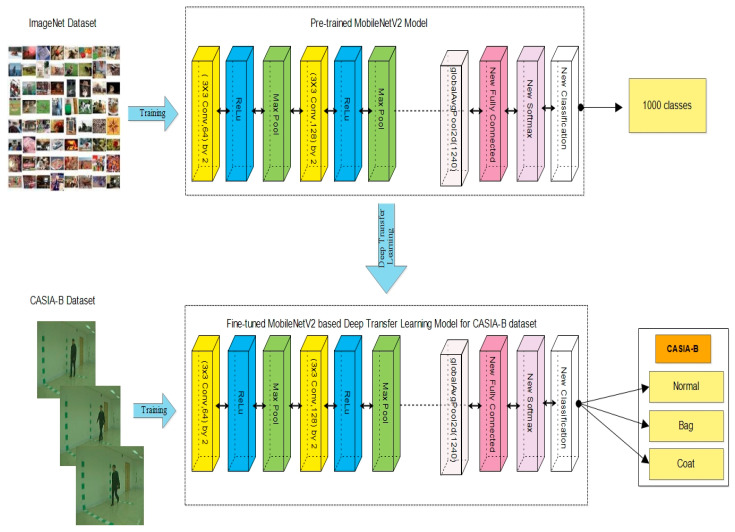
Visual illustration of deep feature extraction using deep transfer learning.

**Task.** The task can be represented as:

(14) ℑ={ℒ, ʄ(.)}

which includes two factors: the label space, ℒ, and a mapping function, ʄ(.), where ℒ={L | Li ℇ ℒ, I=1,……….M}, and M is a label set for the relevant instance in D. The mapping function, ʄ(.), generally known as ʄ(V)=ρ (V|L), is a non-linear and indirect function that may fill the gap between input instances as well as the projected judgment that is acquired from the suggested datasets. Similarly, distinct objectives are specified because of the label spaces among these tasks. The visual process of deep transfer learning is shown in Figure 5. This figure illustrates that none of the layers are frozen, and the entire network is trained on the selected enhanced CASIA-B dataset. After training, features are extracted from the selected layers, such as global average pooling. In the first trained model, named MobilenetV2, the global average pooling layer is employed and performed the activation. From this layer, we obtained 1280 features for each image. Hence, the results vector is denoted by N×1280. In the second trained model, named ShuffleNet, we extracted features from the global average pooling layer and obtained 544 features; hence, the resultant vector is obtained on dimensional N×544. This is shown in Table 2.

### 2.4. Novelty 2: Minimal Serial Features Fusion

The two feature vectors FV1 and FV2 contain N×1280 and N×544 features for MobilenetV2 and ShuffleNet models, respectively. FV3 is a fused feature vector using a serial approach that returns a feature vector of dimension N×1824 by employing the following mathematical formulation:

(15) $$FV3={\left(\begin{array}{c} FV1\\ FV2 \end{array}\right)}_{\left(N\times 1280+N\times 544\right)}$$

The resultant fused feature vector consists of some redundant information; therefore, we implemented a minimization function that removes the redundant features after each iteration. The objective function of this function is to minimize the error rate and reduce the computational time that is required after the reduction of the redundant features. Mathematically, this function is defined as follows:

$$F=\underset{FV3}{\arg\min}\left(Er\right)$$

Also, the working of this process is described in the below Algorithm 1, and the final fused vector is denoted by $\tilde{FV}$.

**Algorithm 1:** Proposed Feature Fusion.**Input:** Feature Vectors ←FV1 and FV2
**Step 1:** Serially Fused using below Equation
   $FV3={\left(\begin{array}{c} FV1\\ FV2 \end{array}\right)}_{\left(N\times 1280+N\times 544\right)}$
**Step 2:** for I = 1 to sizeof(FV3)
**Step 3:** Initialize the static parameter
   Er = 0
   Iterations = 100
**Step 4:** Minimization Function
   $F=\underset{FV3}{\arg\min}\left(Er\right)$
   if(F > Er)
   Repeat above steps
   Stop when Er near to Zero or Equal to Zero
**Output:** Final Fused Feature Vector $\leftarrow \tilde{FV}$

### 2.5. Novelty 3: Proposed ESOcNR Feature Selection

Feature selection has been an important step in machine learning over the last two years. Many techniques have been introduced, but they faced a few issues, such as reducing important features and selecting extra features. These factors can reduce the accuracy and increase the computational time. This work proposed a new equilibrium state optimization technique controlled using the Newton–Raphson method (ESOcNR) for the best feature selection. The proposed technique is initially based on the original ESO algorithm [28], which uses a mass balance equation to define the concentration of a nonreactive ingredient in a control volume. The mass balance equation describes the mechanics of mass entering, leaving, and creating mass in a control volume. The universal mass-balance equation is represented by a first-order ordinary differential equation, described as follows:

(16) $$W\frac{dE}{dt}=RE_{fr}-RD+H$$

where W represents the inside of the control volume, D denotes the concentration, the rate of mass change in the control volume is denoted by $W\frac{dE}{dt}$, and R denotes the volumetric efficiency of a control volume. The variable $E_{fr}$ implies the concentration at an equilibrium state with no production within the control volume, and H denotes the mass generation rate within the control volume. A stable equilibrium condition is achieved once $W\frac{dE}{dt}$ hits zero. Reordering of the above equation helps in solving $\frac{dE}{dt}$ as a function of $\frac{R}{W}$, where $\frac{R}{W}$ reflects the inverse of the residency period, referred as *λ* or the turnover rate $\left(\lambda =\frac{R}{W}\right)$. The W is computed as follows:

(17) $$W=\frac{dE}{\lambda E_{fr}-\lambda E+\frac{H}{W}}$$

(18) $$W=\int_{E_{0}}^{E}\frac{dE}{\lambda E_{fr}-\lambda E+\frac{H}{W}}$$

(19) $$E=E_{fr}+(E_{0}-E_{fr})G+\frac{H}{\lambda W}\left(1-G\right)$$

In the above equation, the H is determined as follows:

(20) $$H=\exp\left[-\lambda \left(t-t_{0}\right)\right]$$

where $t_{0}$ and $E_{0}$ are the initial start time and concentration and are calculated by an integral interval, respectively. Equation (19) can be utilized to determine the attention in the control volume with a specified turnover rate, among several other things. It can also be employed to compute the average turnover rate by implementing a simple linear regression with a predetermined generation rate.

The first term is equilibrium concentration, which is one of the ideal solutions chosen randomly from a pool known as the equilibrium pool. The direct search approach is the second term concerned primarily with a concentration difference between a particle and the equilibrium state. The said term acts as an explorer, urging particles to explore the entire region. The third term is associated with the generation rate that either primarily contributes as an exploiter or remedy refiner, but can also function as an explorer occasionally. Each term is defined below and how it influences the search pattern.

Evaluation and Initialization of Functions: The optimization process is initiated by ESO, as are several other meta-heuristic algorithms with the initial population. The dimensions of the search space with uniform random initialization and initial concentrations are determined by the number of particles as follows:

(21) $$E_{i}^{initial}=E_{min}+rand_{i}\left(E_{max}-E_{min}\right)i=1,2,3\ldots \ldots n$$

The ith particle’s initial concentration vector is indicated by $E_{i}^{initial}$, while the maximal and minimal values for the dimensions are given by $E_{max}$ and $E_{min}$, respectively. The variable n is the population’s number, and $rand_{i}$ is a haphazard vector that falls inside the range of 0–1. To evaluate the equilibrium candidates for the fitness function, particles are appraised and then classified.

Candidates and the Equilibrium Pool $E_{\mathrm{fr}}$: The algorithm’s ultimate convergence state is the global optimal equilibrium state. There is no knowledge about the equilibrium state at the outset of the optimization procedure; thus, only equilibrium candidates are picked to create a search pattern for the particles. The finest four particles are discovered throughout the optimization process, as well as one additional particle whose concentration matches the arithmetic mean of the four previously mentioned particles. These four options aid EO in improving its exploring abilities, whereas the average helps with exploitation. The number of candidates is arbitrary and determined by the nature of the optimization issue.

In contrast, selecting fewer than four candidates degrades the method performance in multimodal and composition functions, although improving outcomes in uni-modal functions. Furthermore, having more than four candidates may have a detrimental effect. Thus five particles are referred to as equilibrium candidates and are utilized to form the equilibrium pool vector:

(22) $${\vec{E}}_{eq\_pool}=\left\{{\vec{E}}_{eq\left(1\right)},{\vec{E}}_{eq\left(2\right)},{\vec{E}}_{eq\left(3\right)},{\vec{E}}_{eq\left(4\right)},{\vec{E}}_{eq\left(ave\right)}\right\}$$

Each particle’s concentration is changed at random in each cycle by picking randomly from a pool of candidates with the same probability. For instance, in the first iteration, the first particle upgrades concentrations based on ${\vec{E}}_{eq\left(1\right)}$; in the second iteration, concentrations may be updated based on ${\vec{E}}_{eq\left(ave\right)}$. Each particle will be updated until the optimization process is complete, with about the same percentage of updates going to each candidate solution.

Exponential Term (H): The next term which further contributes to the primary concentration update rule is the exponential term (H). A thorough explanation of such a concept might help EO strike a fair equilibrium between exploration and exploitation. Because the turnover rate in a real control volume fluctuates over time, λ is a random vector that tends to range from 0–1.

(23) $$\vec{H}=e^{-\vec{\lambda }\left(t-t_{0}\right)}$$

The time is represented by t and formulated as follows:

(24) $$t={\left(1-\frac{Itr}{Max\_Itr}\right)}^{\left(b_{2}\frac{Itr}{Max\_Itr}\right)}$$

where Itr and Max_Itr represent the current and max number of iterations, respectively, and $b_{2}$ is a constant used to adjust exploitation capabilities. To ensure convergence, the search velocity is reduced to increasing the algorithm’s exploration and exploitation capabilities.

(25) $${\vec{t}}_{0}=\frac{1}{\vec{\lambda }}\ln\left(-b_{1}sign\left(\vec{s}-0.5\right)\left[1-e^{-\vec{\lambda }t}\right]\right)+t$$

where, $b_{1}$ is a constant number that regulates exploration. The higher $b_{1}$ value, the better the exploring capacity, and hence the lower the exploitation efficiency. Similarly, increasing $b_{2}$ improves exploitation while decreasing exploring capabilities. The third component sign $\left(\vec{s}-0.5\right)$ influences the course of exploration and exploitation. The variable $\vec{s}$ is a random vector with a value ranging from 0 to 1. These constants are calculated empirically by evaluating a set of test functions. These parameters, however, can be modified as needed for specific circumstances.

(26) $$\vec{H}=b_{1}sign\left(\vec{s}-0.5\right)\left[e^{-\vec{\lambda }t}-1\right]$$

Generation Rate (G): The generating rate is one of the most important words in providing the proper answer by improving the exploitation phase. The model below displays generation rates as a first-order exponential decay process:

(27) $$\vec{G}={\vec{G}}_{0}e^{-\vec{l}\left(t-t_{0}\right)}$$

where ${\vec{H}}_{0}$ represents the beginning value and $\vec{l}$ represents the decay constant. To have a more controlled and systematic search pattern and to limit the number of random variables, we assume $\vec{l}$ = $\vec{\lambda }$ and use the previously computed exponential term. As a result, the final set of generation rate equations are as follows:

(28) $$\vec{G}={\vec{G}}_{0}\vec{H}$$

(29) $$\vec{H}=e^{-\vec{\lambda }\left(t-t_{0}\right)}$$

(30) $${\vec{G}}_{0}=\vec{GCP}\left(\vec{D_{eq}}-\vec{\lambda }\vec{D}\right)$$

(31) $$\vec{GCP}=\{\begin{array}{c} 0.5s_{1}\;s_{2}\geq GP\\ 0\;s_{2}\geq GP \end{array}$$

The generation rate control parameter (GCP) is a generic term for the potential contribution towards the updating process. However, one component (called the generation probability (GP)) determines the likelihood of this contribution by defining how many particles utilize the generic term to update their states. By using GP = 0.5, a reasonable balance between exploration and exploitation may be reached. Finally, the EO update regulation is defined as follows:

(32) $$\vec{E}={\vec{E}}_{eq}+\left(\vec{E}-{\vec{E}}_{eq}\right)\times \vec{H}+\frac{\vec{G}}{\vec{\lambda }W}\left(1-\vec{H}\right)$$

The first term reflects an equilibrium concentration, whereas the second and third terms describe variations in concentration. The second term is in charge of searching the entire area for the best position. The third term contributes to exploitation by making the solution more exact when it reaches a spot. Depending on factors, such as particle concentrations, equilibrium candidates, and the turnover rate (λ), the second and third components may have the same or opposite sign. The same sign promotes diversity, which helps with domain searches, while the opposite sign minimizes variation, which helps with local searches. Finally, the memory-saving algorithms help each particle maintain track of its locations in space, which influences its fitness value. Each particle’s fitness value in the current iteration is assessed to the one from the previous iteration and is rewritten if it attains a better choice. This process improves exploitation capacity but increases the likelihood of being caught in local minima if the approach does not benefit from global exploration capability.

Newton–Raphson based Final Selection: The features of each iteration of ESO are passed to the Newton-Raphson-based function [29] that computes the resultant value. The resultant value states when the number of iterations will be stopped. The main purpose of this function is to find the quick value for the threshold selection. This value reduces the computational time and improves the performance. Mathematically, this process is defined as follows:

(33) $$f_{1}=f_{0}-\frac{h\left(f_{0}\right)}{h\prime \left(f_{0}\right)}$$

(34) $$f_{n+1}=f_{n}-\frac{h\left(f_{n}\right)}{h\prime \left(f_{n}\right)}$$

where, $f_{n+1}\in \vec{E}$ and is a selected feature vector after each iteration. This vector is updated after each iteration, and once the value of $f_{n+1}$ becomes constant, it will stop and return the best feature vector. The final selected feature is finally classified using machine learning classifiers.

## 3. Results and Analysis

Dataset and Performance Measures: The proposed HGR framework has been evaluated using the CASIA-B dataset. A detailed description of a dataset has been given in Section 3. Several classifiers have been used for classification accuracies, such as fine tree, medium tree, linear SVM, quadratic SVM, weighted KNN, coarse KNN, bagged trees, subspace discriminate, Bi-layered neural network, and Tri-layered neural network. The performance of each classifier is computed using recall rate, precision rate, accuracy, and time (seconds).

Experimental Setup: We divided the entire dataset 50:50 for training and testing. The 10-fold cross-validation was chosen for the testing process. Moreover, several hyperparameters have been utilized in training deep learning models, such as a learning rate of 0.0001, epochs are 100, momentum value of 0.7, mini-batch size of 32, and the chosen optimizer was the stochastic gradient descent. The entire framework was simulated on MATLAB 2022a using a personal computer Corei7 having 16 GB of RAM and 8 GB graphics card.

### 3.1. Results Analysis

Table 3 presents the results of angle 0 of the CASIA-B dataset using the proposed framework. The results of the fusion and optimization methods are presented in this table. Several classifiers have been employed for the classification results. First, the fusion method obtained the highest accuracy of 97.3% on Quadratic SVM, whereas the recall rate is 97.33% and the precision rate was 97.37%. Computational time was also noted, and it was observed that the fusion process consumes 572.19 s for this classifier. However, the minimum noted time for the fusion process was 69.438 s on the medium tree classifier, whereas the maximum reported time was 4420.7 s on the tri-layered neural network classifier. Second, the optimization results have been presented and obtained the maximum accuracy of 97.2% on Quadratic SVM. The recall rate of this classifier was 97.23%, and the precision rate was 97.3%. The computation time of this step was also noted, and Quadratic SVM executes in 45.259 s. However, the minimum noted time for the optimization process is 38.502 s on the medium tree classifier, whereas the maximum reported time was 2049.1 s on the tri-layered neural network classifier. This shows that the accuracy of the optimization process was almost consistent, but the execution time was significantly reduced, which thus shows the strength of the proposed framework.

Table 4 presents the results of the fusion and optimization methods on angle 18 of the CASIA-B dataset using the proposed framework. First, the fusion method obtained the highest accuracy of 98.6% on Quadratic SVM, whereas the recall rate was 98.57% and the precision rate was 98.57%. Computational time is also noted, and it was observed that the fusion process consumes 859.46 s for this classifier. However, the minimum noted time for the fusion process was 174.72 s on the fine tree classifier, whereas the maximum reported time was 2049.8 s on the subspace discriminant classifier. Second, the optimization obtained a maximum accuracy of 98.0% on Quadratic SVM. The recall rate of this classifier was 97.93%, and the precision rate was 98%. The computation time of this step was also noted, and Quadratic SVM executes in 42.512 s. However, the minimum noted time for the optimization process was 34.35 s on the medium tree classifier, whereas the maximum reported time is 1573.6 s on the bagged trees classifier. This shows that the accuracy of the optimization process was almost consistent, but the execution time was significantly reduced.

Table 5 presents the results of angle 36 of the CASIA-B dataset using the proposed framework. First, the fusion method obtained the highest accuracy of 97.7% on Quadratic SVM, whereas the recall rate was 97.67% and the precision rate was 97.63%. Computational time was also noted, and observed that the fusion process consumes 1014.7 s for this classifier. However, the minimum noted time for the fusion process was 95.118 s on the medium tree classifier, whereas the maximum reported time was 5675.8 s on the bagged trees classifier. Second, the optimization obtained the maximum accuracy of 97.2% on Quadratic SVM. The recall rate of this classifier was 97.23%, and the precision rate was 97.23%. The computation time of this step was also noted, and Quadratic SVM executes in 53.864 s. However, the minimum noted time for the optimization process was 23.989 s on the medium tree classifier, whereas the maximum reported time was 1827.5 s on the bagged trees classifier. Results in this table show the consistency, but time was significantly reduced, which is a main strength of this step.

Table 6 discusses the results of angle 54 of the CASIA-B dataset using the proposed framework. First, the fusion method obtained the highest accuracy of 96.5% on Quadratic SVM, whereas the recall rate was 96.53% and the precision rate was 96.53%. The minimum noted computational time for the fusion process was 45.349 s on the medium tree classifier, whereas the maximum reported time was 4780.4 s on the bagged trees classifier. Second, the optimization obtained the maximum accuracy of 96.2% on Quadratic SVM. The recall rate of this classifier was 96.27%, and the precision rate was 96.27%. After this process, the computational time was significantly reduced, but not much change occurred in the accuracy.

Table 7 presents the results of angle 72 of the CASIA-B dataset using the proposed framework. First, the fusion method obtained the highest accuracy of 92.8% on coarse KNN, whereas the recall rate was 82.87% and the precision rate was 82.97%. Computational time was also noted; the minimum noted time was 232.76 s on the medium tree classifier, whereas the maximum reported time was 3645.4 s on the tri-layered neural network classifier. Second, the optimization obtained the maximum accuracy of 92.9% on coarse KNN. The recall rate of this classifier was 83%, and the precision rate was 82.9%. The minimum computation time of this step was 72.293 s on the medium tree classifier, whereas the maximum reported time was 2918 s on the tri-layered neural network classifier. Overall, this step reduced the computational time and remained consistent with the classification accuracy.

Table 8 shows the results of angle 90 of the CASIA-B dataset using the proposed framework. Results are presented for both the fusion and optimization steps. First, the fusion method obtained the highest accuracy of 93.7% on Quadratic SVM, whereas the recall rate was 93.73% and the precision rate was 94.03%. The minimum noted computational time on the medium tree classifier was 281.13 s, whereas the maximum reported time was 4960.7 s on the bagged trees classifier. Second, the optimization obtained the maximum accuracy of 93.1% on Quadratic SVM. The recall rate of this classifier was 93.17%, and the precision rate was 93.53%. The minimum recorded time for the optimization process was 78.005 s on the medium tree classifier, whereas the maximum reported time was 1280.4 s on the weighted KNN classifier. These facts show that the accuracy was not changed too much, but a significant reduction was noted in computational time, which is the strength of the proposed optimization algorithm.

Table 9 presents the results of angle 108 of CASIA-B dataset using the proposed framework. In this table, the fusion process obtained the highest accuracy of 94.7% on Quadratic SVM, whereas the recall rate was 94.57% and the precision rate was 94.63%. Second, the optimization process obtained the maximum accuracy of 94.2% on Quadratic SVM. Computational time was noted for both experiments and the minimum noted time for the fusion process was 83.087 s on the medium tree classifier. In contrast, the minimum noted time for the optimization process was 39.314 s on the medium tree classifier. This shows the significant improvement in the optimization process’s computational time, which is the strength of this step.

Similarly, Table 10 presents the results of angle 126 of the CASIA-B dataset using proposed framework. First, the fusion method obtained the highest accuracy of 91.2% on Quadratic SVM, whereas the recall rate was 91.13% and precision rate was 91.17%. Computational time was also computed, and the minimum noted time for the fusion process was 346.5 s on the medium tree classifier. Second, the optimization results have been presented and obtained the maximum accuracy of 90.4% on Quadratic SVM. The minimum computational time of this step was 25.116 s on the fine tree classifier. Hence, it is clearly observed that the proposed framework improved the accuracy and reduced the computational time.

Table 11 presents the results of angle 144 of CASIA-B dataset using the proposed framework. In this table, the fusion method obtained the highest accuracy of 92.4% on Quadratic SVM, whereas the recall rate is 92.34% and the precision rate is 92.6%. The minimum computational time for this experiment is 126.29 s on the fine tree classifier. Second, the optimization results have been presented and obtained the maximum accuracy of 91.9% on Quadratic SVM. The recall rate of this classifier is 91.9%, and the precision rate is 92.17%. Compared to the fusion process, the computation time of this step has been significantly reduced to 96.952 s on the fine-tree classifier.

Table 12 presented the results of angle 162 of the CASIA-B dataset and obtained the maximum accuracy for the fusion method was 96.5% on Quadratic SVM. In contrast, the recall rate was 96.47%, and the precision rate was 96.57%. Computational time was also noted, and it was observed that the minimum reported time was 32.703 s on the medium tree classifier. Second, the optimization obtained the maximum accuracy of 96.3% on Quadratic SVM. The recall rate of this classifier was 96.23%, and the precision rate was 96.4%. The computation time of this step was 68.363 s on the fine-tree classifier, which is significantly lower that the fusion process.

Table 13 presents the results of angle 180 of CASIA-B dataset using the proposed framework. First, the fusion method obtained the highest accuracy of 99.9% on Quadratic SVM, whereas the recall rate was 99.83% and the precision rate was 99.87%. Computational time was also noted, and observed that the fusion process consumes 240.29 s for this classifier. However, the minimum noted time for the fusion process was 88.322 s on the medium tree classifier, whereas the maximum reported time was 2476.1 s on the bagged trees classifier. Second, the optimization obtained the maximum accuracy of 99.8% on Quadratic SVM. The recall rate of this classifier was 99.8%, and the precision rate was 99.77%. The computation time of this step was also noted, and Quadratic SVM executes in 113.25 s. However, the minimum noted time for the optimization process was 28.024 s on the bi-layered neural network classifier, whereas the maximum reported time was 168.63 s on the weighted KNN classifier. This shows that the accuracy of the optimization process was almost consistent, but the execution time was significantly reduced.

### 3.2. Comparative Analysis

**Figure 6:**
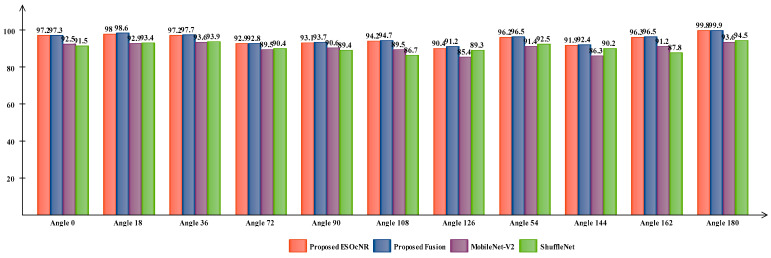
Visual analysis of intermediate steps of the proposed framework.

**Figure 7:**
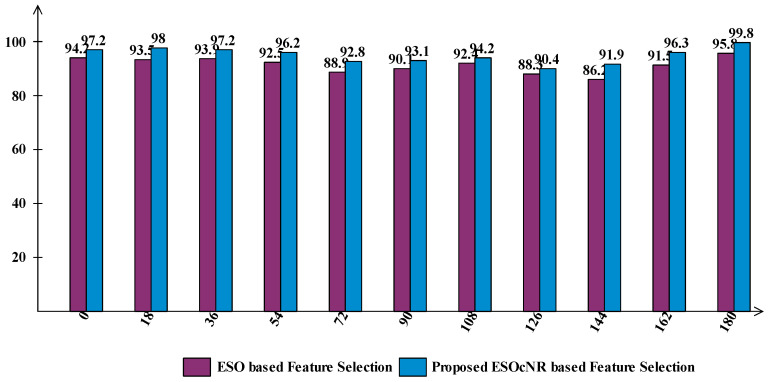
Comparison of proposed ESOcNR feature selection algorithm with original ESO based selection.

A detailed comparison of the proposed framework has been included in this section based on the intermediate steps’ performance and individual ESO-based feature selection. Figure 6 shows the analysis of the intermediate steps of the proposed framework. This figure illustrates that the accuracy of the ShuffleNet and MobilenetV2 deep model features is insufficient, and both models performed better for a few angles. However, the fusion process improves the accuracy, but an increase occurred in the computational time, as presented in Table 3, Table 4, Table 5, Table 6, Table 7, Table 8, Table 9, Table 10, Table 11, Table 12 and Table 13. Therefore, a new technique named ESOcNR is proposed. Using this technique, a significant reduction occurred in the number of features, but a minor drop occurred in accuracy (Table 3, Table 4, Table 5, Table 6, Table 7, Table 8, Table 9, Table 10, Table 11, Table 12 and Table 13). Figure 7 illustrates the comparison between ESO-based feature selection and ESOcNR-based feature selection. In this figure, it is noted that the accuracy was improved after employing the proposed selection method. For example, for angle 0, a 3% change occurred; however, for the other angles, an almost 3–4% change is reported after employing the proposed ESOcNR. Table 14 shows the results of the proposed feature selection technique for CASIA-B dataset on all 11 angles. In this table, it is noted that the QSVM classifier shows the most improved accuracy for the most angles. Table 15 presents the comparison of the proposed framework with state-of-the-art techniques. In this table, the comparison is conducted with each angle of the CASIA-B dataset. The proposed framework performed better on angles 0, 18, 36, 54, 144, 162, and 180. However, the performance on other angles (72, 90, 108, and 126) is not improved, which will be considered in the future. Hence, overall, the proposed framework shows improved accuracy.

## 4. Conclusions

A new framework is proposed in this work based on fusion-assisted deep learning features and the ESOcNR feature selection technique. The proposed framework consists of a few important subsequent steps, including contrast enhancement of video frames, deep learning features extraction from the selected models, proposed minimal serial fusion approach, and ESOcNR-based feature selection. Results are computed on the enhanced CASIA-B dataset using all 11 angles and show improvements in accuracy on 7 of these 11 angles. Based on the results and comparative analysis, we conclude the following points:

- The training of deep learning models on the enhanced dataset has extracted the more useful features that later improved the accuracy.
- The proposed fusion approach improved the accuracy but increased the computation time.
- The original ESO-based feature selection approach selected some redundant features that reduced the classification accuracy.
- Selection of best features using the proposed ESOcNR maintains the classification accuracy and reduces the computational time of the fusion process.

In the future, the weights of deep learning models can be optimized using some meta-heuristic features selection techniques. Moreover, in the future, the angles 72, 90, 108, and 126 should be analyzed to attempt to improve their accuracy.

## Figures and Tables

**Table 1:** Description of the extracted dataset (CASIA-B) on all selected angles.

CASIA-B Dataset		Before Augmentation No. of Images	After Augmentation No. of Images
**0**	Bag	2347	9388
	Coat	2436	9744
	Normal	2191	8764
18	Bag	2457	9828
	Coat	2428	9712
	Normal	2550	10,200
36	Bag	2459	9836
	Coat	2530	10,120
	Normal	2285	9140
54	Bag	2578	10,312
	Coat	2661	10,644
	Normal	2433	9732
72	Bag	2531	10,124
	Coat	2582	10,328
	Normal	2471	9884
90	Bag	2512	10,048
	Coat	2717	10,868
	Normal	2320	9280
108	Bag	2363	9452
	Coat	2753	11,012
	Normal	2647	10,588
126	Bag	2472	9888
	Coat	2301	9204
	Normal	2418	9672
144	Bag	2436	9744
	Coat	2452	9808
	Normal	2438	9752
162	Bag	2466	9864
	Coat	2528	10,112
	Normal	2422	9688
180	Bag	2423	9692
	Coat	2674	10,696
	Normal	2625	10,500

**Table 2:** Description of deep features for the selected deep models.

Model	Layer	Input	Output	Feature Vector
Fine-tuned MobilenetV2	154	224 × 224 × 3	1280	N × 1280
Fine-tuned ShuffleNet	172	224 × 224 × 3	544	N × 544

**Table 3:** Classification results of HGR using proposed framework on angle 0 of the CASIA-B dataset.

Classifiers	Features		Recall (%)	Precision (%)	Accuracy (%)	Time (s)
	Fusion	Optimization				
Fine Tree	✔		94.8	94.83	94.8	154.08
		**✔**	94.63	94.63	94.6	61.748
Medium Tree	✔		95.17	95.2	95.1	69.438
		✔	93	93.2	93.0	38.502
Linear SVM	✔		97.3	97.3	97.2	466.93
		✔	97.17	97.2	97.1	192.66
Quadratic SVM	✔		97.33	97.37	**97.3**	572.19
		✔	97.23	97.3	**97.2**	45.259
Coarse KNN	✔		96.17	96.23	96.1	1290.8
		✔	96.37	96.4	96.3	585.42
Weighted KNN	✔		96.27	96.27	96.2	1347.6
		✔	96.1	96.1	96.0	656.35
Bagged Trees	✔		95.6	95.6	95.5	3765.9
		✔	95.33	95.3	95.2	1422.3
Subspace Discriminant	✔		96.97	97	96.9	2999.2
		✔	96.6	96.67	96.6	575.46
Bilayered Neural Network	✔		96.07	96.07	96.0	4155.3
		✔	95.8	95.83	95.8	2032.2
Trilayered Neural Network	✔		96.3	96.27	96.2	4420.7
		✔	95.7	95.6	95.6	2049.1

**Table 4:** Classification results of HGR using the proposed framework on angle 18 of the CASIA-B dataset.

Classifiers	Features		Recall (%)	Precision (%)	Accuracy (%)	Time (s)
	Fusion	Optimization				
Fine tree	✔		92.97	93	92.9	174.72
		**✔**	92.03	92.07	92.0	48.364
Medium tree	✔		91.17	91.37	91.2	505.02
		✔	89.37	89.57	89.4	34.35
Linear SVM	✔		98.4	98.37	98.4	498.59
		✔	97.77	97.77	97.7	204.978
Quadratic SVM	✔		98.57	98.57	**98.6**	859.46
		✔	97.93	98	**98.0**	42.512
Coarse KNN	✔		94.93	95.23	95.0	873.62
		✔	95.4	95.5	95.4	691.91
Weighted KNN	✔		96.93	97.07	96.9	695.55
		✔	96.87	97	96.9	668.99
Bagged trees	✔		96	96.03	96.0	2899.7
		✔	95.27	95.27	95.3	1573.6
Subspace discriminant	✔		97.57	97.52	97.5	2049.8
		✔	96.5	96.57	96.5	621.48
Bilayered neural network	✔		98.23	98.20	98.2	726.05
		✔	97.07	97.07	97.1	839.48
Trilayered neural network	✔		98.27	98.27	98.3	600.06
		✔	97.17	97.2	97.2	1216.1

**Table 5:** Classification results of HGR using proposed framework on angle 36 of CASIA-B dataset.

Classifiers	Features		Recall (%)	Precision (%)	Accuracy (%)	Time (s)
	Fusion	Optimization				
Fine tree	✔		90.17	90.23	90.2	175.99
		**✔**	89.57	89.57	89.6	30.051
Medium tree	✔		87.4	87.33	87.4	95.118
		✔	86.8	86.83	86.8	23.989
Linear SVM	✔		97.43	97.37	97.4	850.09
		✔	96.83	96.8	96.8	259.84
Quadratic SVM	✔		97.67	97.63	**97.7**	1014.7
		✔	97.23	97.23	**97.2**	53.864
Coarse KNN	✔		94.3	94.23	94.3	2152.5
		✔	93.8	93.73	93.8	699.39
Weighted KNN	✔		95.67	95.57	95.6	2562.8
		✔	95.13	95.07	95.1	671.14
Bagged trees	✔		95.17	95.13	95.1	5675.8
		✔	93.7	93.7	93.7	1827.5
Subspace discriminant	✔		96.6	96.57	96.6	3608.6
		✔	95.07	95.03	95.1	805.07
Bilayered neural network	✔		97.17	97.17	97.2	2644.7
		✔	96.07	96.07	96.1	1054
Trilayered neural network	✔		97.1	97.1	97.1	3075.3
		✔	95.67	95.7	95.7	1131.5

**Table 6:** Classification results of HGR using the proposed framework on angle 54 of CASIA-B dataset.

Classifiers	Features		Recall (%)	Precision (%)	Accuracy (%)	Time (s)
	Fusion	Optimization				
Fine tree	✔		90	90	90.0	126.75
		**✔**	89.53	89.6	89.5	108.46
Medium tree	✔		87	87	86.9	45.349
		✔	86.2	86.03	86.0	32.89
Linear SVM	✔		96.17	96.13	96.1	835.99
		✔	95.77	95.8	95.7	290.16
Quadratic SVM	✔		96.53	96.53	**96.5**	1020
		✔	96.27	96.27	**96.2**	320.93
Coarse KNN	✔		93.23	93.47	93.2	1881.1
		✔	93.37	93.57	93.4	652.75
Weighted KNN	✔		95.13	95.13	95.1	1889.2
		✔	95	95.03	95.0	913.86
Bagged trees	✔		94.3	94.37	94.3	4780.4
		✔	93.63	93.67	93.6	1510.8
Subspace discriminant	✔		95.67	95.67	95.7	3385
		✔	94.67	94.7	94.7	863.8
Bilayered neural network	✔		96.13	96.13	96.2	2521
		✔	95.33	95.33	95.3	923.33
Trilayered neural network	✔		96	96	96.0	2438.8
		✔	95.03	95	95.0	1013.3

**Table 7:** Classification results of HGR using the proposed framework on angle 72 of the CASIA-B dataset.

Classifiers	Features		Recall (%)	Precision (%)	Accuracy (%)	Time (s)
	Fusion	Optimization				
Fine tree	✔		8.87	80.97	90.8	300.21
		**✔**	80.47	80.53	80.4	107.03
Medium tree	✔		75.7	75.7	75.6	232.76
		✔	76.3	78.57	76.1	72.293
Linear SVM	✔		86.93	87.17	86.9	1263.8
		✔	86	86.17	85.9	666.27
Quadratic SVM	✔		87.6	87.57	87.5	1588.8
		✔	86.93	86.87	86.8	204.15
Coarse KNN	✔		82.87	82.97	**92.8**	1324.9
		✔	83	82.9	**92.9**	791.35
Weighted KNN	✔		85.8	85.77	85.7	1369.1
		✔	85.17	85.17	85.1	925.01
Bagged trees	✔		85.03	85.07	85.0	2694.2
		✔	84.87	84.87	84.7	2772.8
Subspace discriminant	✔		86.67	86.97	86.6	2415.2
		✔	85.37	85.53	85.3	1544.1
Bilayered neural network	✔		85.57	85.57	85.0	3259.8
		✔	84.1	84.1	84.0	2850.5
Trilayered neural network	✔		85.5	85.57	85.4	3645.4
		✔	84.43	84.47	84.4	2918

**Table 8:** Classification results of HGR using the proposed framework on angle 90 of the CASIA-B dataset.

Classifiers	Features		Recall (%)	Precision (%)	Accuracy (%)	Time (s)
	Fusion	Optimization				
Fine tree	✔		88	88.43	88.0	1773.7
		**✔**	87.8	88.43	87.8	104.61
Medium tree	✔		85.37	86.5	85.4	281.13
		✔	83.5	84.57	83.3	78.005
Linear SVM	✔		92.73	93.33	92.7	1871.9
		✔	92.4	92.9	92.4	735.43
Quadratic SVM	✔		93.73	94.03	**93.7**	1865.3
		✔	93.17	93.53	**93.1**	737.25
Coarse KNN	✔		89.57	90.33	89.4	2268.8
		✔	89.33	90.13	89.2	553.79
Weighted KNN	✔		92.4	92.57	92.4	2467.2
		✔	92.07	92.13	91.9	1280.4
Bagged trees	✔		91.67	91.79	91.7	4960.7
		✔	91.5	91.73	91.5	1041.7
Subspace discriminant	✔		92.47	93.33	92.5	4581.6
		✔	90.93	92.07	91.0	557.85
Bilayered neural network	✔		92.67	92.67	92.6	3507.9
		✔	91.5	91.53	91.4	808.15
Trilayered neural network	✔		92.9	92.93	92.8	4274.3
		✔	91.47	91.47	91.4	1225

**Table 9:** Classification results of HGR using the proposed framework on angle 108 of CASIA-B dataset.

Classifiers	Features		Recall (%)	Precision (%)	Accuracy (%)	Time (s)
	Fusion	Optimization				
Fine tree	✔		89.1	89.1	89.2	195.8
		**✔**	88.03	88.1	88.2	60.125
Medium tree	✔		83.8	84.5	84.3	83.087
		✔	82.5	85	83.2	39.314
Linear SVM	✔		94.3	94.33	94.4	1126.1
		✔	93.6	94.13	93.7	276.45
Quadratic SVM	✔		94.57	94.63	**94.7**	1382
		✔	94.1	94.13	**94.2**	268.99
Coarse KNN	✔		89.93	89.9	90.0	1970
		✔	90	89.97	90.1	418.15
Weighted KNN	✔		92.57	95.6	92.7	2148.8
		✔	92.23	92.27	92.3	1020.5
Bagged trees	✔		93.3	93.33	93.4	4736.1
		✔	92.8	92.8	92.9	1467.7
Subspace discriminant	✔		93.87	93.97	94.0	3994.2
		✔	92.8	92.97	93.0	426.27
Bilayered neural network	✔		93.87	93.9	93.9	3528.1
		✔	92.6	92.6	92.7	1079
Trilayered neural network	✔		93.63	93.67	93.7	3639.6
		✔	92.53	92.57	92.6	1315.2

**Table 10:** Classification results of HGR using proposed framework on angle 126 of CASIA-B dataset.

Classifiers	Features		Recall (%)	Precision (%)	Accuracy (%)	Time (s)
	Fusion	Optimization				
Fine tree	✔		84.87	85	84.8	468.07
		**✔**	83.9	83.93	83.9	25.116
Medium tree	✔		83.03	85.07	82.8	346.5
		✔	81.9	84.03	81.7	65.492
Linear SVM	✔		90.87	90.9	90.9	1243.8
		✔	89.73	89.7	89.7	254.83
Quadratic SVM	✔		91.13	91.17	**91.2**	1450.2
		✔	90.33	90.37	**90.4**	338.84
Coarse KNN	✔		87.27	87.63	87.3	1743.2
		✔	87.83	88	87.9	359.53
Weighted KNN	✔		89.3	89.37	89.3	1733
		✔	88.37	88.43	88.4	399.24
Bagged trees	✔		89	89.07	89.1	3541.7
		✔	88.5	88.5	88.6	1202.6
Subspace discriminant	✔		90.4	90.47	90.4	2676.8
		✔	88.9	89.17	88.9	705.52
Bilayered neural network	✔		90.23	90.23	90.3	2448.2
		✔	88.4	88.4	88.4	1268.9
Trilayered neural network	✔		90.23	90.2	90.3	3238.2
		✔	88.5	88.5	88.6	1229.1

**Table 11:** Classification results of HGR using the proposed framework on angle 144 of the CASIA-B dataset.

Classifiers	Features		Recall (%)	Precision (%)	Accuracy (%)	Time (s)
	Fusion	Optimization				
Fine tree	✔		87.43	87.47	87.4	126.29
		**✔**	86.5	86.6	86.5	96.952
Medium tree	✔		84.33	85.1	84.3	987.03
		✔	84.1	84.83	84.1	173.73
Linear SVM	✔		92.1	92.43	92.1	2679.4
		✔	91.27	91.73	91.3	296.31
Quadratic SVM	✔		92.37	92.6	**92.4**	3202.6
		✔	91.9	92.17	**91.9**	327.22
Coarse KNN	✔		87.93	88.9	87.9	3544.2
		✔	88.47	89.37	88.5	485.17
Weighted KNN	✔		90.47	90.6	90.5	3531.9
		✔	90.43	90.53	90.4	505.14
Bagged trees	✔		90.77	90.8	90.8	7531.4
		✔	90.43	90.37	90.3	1321.3
Subspace discriminant	✔		91.4	91.63	91.4	1477.2
		✔	90.43	90.8	90.4	676.45
Bilayered neural network	✔		91.2	91.2	91.2	10934
		✔	90.17	90.2	90.2	1393.7
Trilayered neural network	✔		91.27	9127	91.3	1674.1
		✔	89.73	89.7	89.7	1460.7

**Table 12:** Classification results of HGR using proposed framework on angle 162 of CASIA-B dataset.

Classifiers	Features		Recall (%)	Precision (%)	Accuracy (%)	Time (s)
	Fusion	Optimization				
Fine tree	✔		92.27	92.3	92.3	52.63
		**✔**	92.33	92.53	92.4	68.363
Medium tree	✔		90.1	90.4	90.1	32.703
		✔	89.27	89.93	89.4	143.29
Linear SVM	✔		96.23	96.4	96.2	342.89
		✔	96	96.13	96.0	55.835
Quadratic SVM	✔		96.47	96.57	**96.5**	432.39
		✔	96.23	96.4	**96.3**	240.25
Coarse KNN	✔		93.8	94.37	93.8	882.19
		✔	93.9	94.4	93.9	353.4
Weighted KNN	✔		95.5	95.57	95.5	807.78
		✔	95.43	95.53	95.4	384.97
Bagged trees	✔		95.47	95.5	95.5	126.7
		✔	94.8	94.8	94.8	1053.6
Subspace discriminant	✔		96	96.07	96.0	1254.6
		✔	95.2	95.37	95.2	553.05
Bilayered neural network	✔		96.03	96.03	96.03	389.06
		✔	95.37	95.37	95.4	798.62
Trilayered neural network	✔		96.1	96.1	96.1	281.9
		✔	95.37	95.33	95.4	848.98

**Table 13:** Classification results of HGR using the proposed framework on angle 180 of CASIA-B dataset.

Classifiers	Features		Recall (%)	Precision (%)	Accuracy (%)	Time (s)
	Fusion	Optimization				
Fine tree	✔		97.5	97.57	97.5	140.6
		**✔**	97.2	97.17	97.2	106.4
Medium tree	✔		96.7	96.7	96.7	88.322
		✔	95.57	95.57	95.6	46.451
Linear SVM	✔		99.87	99.87	99.9	243.19
		✔	99.73	99.73	99.8	20.143
Quadratic SVM	✔		99.83	99.87	**99.9**	240.29
		✔	99.8	99.77	**99.8**	113.25
Coarse KNN	✔		99.33	99.37	99.4	1815.3
		✔	99.07	99.03	99.1	164.76
Weighted KNN	✔		99.73	99.73	99.7	1883.6
		✔	99.6	99.57	99.6	168.63
Bagged trees	✔		99.07	99.1	99.1	2476.1
		✔	98.87	98.87	98.9	338.02
Subspace discriminant	✔		99.63	99.63	99.6	1925.6
		✔	99.47	99.47	99.5	112.38
Bilayered neural network	✔		99.77	99.77	99.8	237.92
		✔	99.7	99.7	99.7	28.024
Trilayered neural network	✔		99.8	99.83	99.8	289.07
		✔	99.67	99.7	99.7	34.53

**Table 14:** Overall proposed feature selection based obtained accuracy for CASIA-B dataset on all 11 angles.

Classifiers	0	18	36	54	72	90	108	126	144	162	180
Fine tree	94.6	92.0	89.6	89.5	80.4	87.8	88.2	83.9	86.5	92.4	97.2
Medium tree	93.0	89.4	86.8	86.0	76.1	83.3	83.2	81.7	84.1	89.4	95.6
Linear SVM	97.1	97.7	96.8	95.7	85.9	92.4	93.7	89.7	91.3	96.0	99.8
Quadratic SVM	**97.2**	**98.0**	**97.2**	**96.2**	86.8	**93.1**	**94.2**	**90.4**	**91.9**	**96.3**	**99.8**
Coarse KNN	96.3	95.4	93.8	93.4	**92.9**	89.2	90.1	87.9	88.5	93.9	99.1
Weighted KNN	96.0	96.9	95.1	95.0	85.1	91.9	92.3	88.4	90.4	95.4	99.6
Bagged trees	95.2	95.3	93.7	93.6	84.7	91.5	92.9	88.6	90.3	94.8	98.9
Subspace discriminant	96.6	96.5	95.1	94.7	85.3	91.0	93.0	88.9	90.4	95.2	99.5
Bilayered NN	95.8	97.1	96.1	95.3	84.0	91.4	92.7	88.4	90.2	95.4	99.7
Trilayered NN	95.6	97.2	95.7	95.0	84.4	91.4	92.6	88.6	89.7	95.4	99.7

**Table 15:** Comparison of the proposed framework accuracy with state-of-the-art techniques.

Method	Angles										
	0	18	36	54	72	90	108	126	144	162	180
[30] 2022	95.2	93.9	-	-	-	-	-	-	-	-	98.2
[31] 2022	97.0	97.9	-	-	97.2	-	-	-	-	-	96.0
[32] 2022	92.1	96.1	-	95.7	-	93	-	-	-	94.87	91.33
[33] 2022	-	-	-	-	98.3	-	-	-	-	94.90	98.6
[34] 2020	-	94.3	93.8	94.7	-	-	-	-	-	-	-
[35] 2019	98.8	95.6	96.3	91.9	94.0	95.2	94.6	95.4	90.4	93.00	95.1
PROPOSED	**97.2**	**98.0**	**97.2**	**96.2**	92.8	93.1	94.2	90.4	**91.9**	**96.3**	**99.8**

## Data Availability

The dataset used in this work is publically available.
